# Rational Design of Self-Healing Tough Hydrogels: A Mini Review

**DOI:** 10.3389/fchem.2018.00497

**Published:** 2018-10-18

**Authors:** Wenda Wang, Ravin Narain, Hongbo Zeng

**Affiliations:** Department of Chemical and Materials Engineering, University of Alberta, Edmonton, AB, Canada

**Keywords:** hydrogels, synthesis, self-healing, tough hydrogels, functional hydrogels

## Abstract

Hydrogels are three-dimensional cross-linked polymer networks which can absorb and retain large amount of water. As representative soft materials with tunable chemical, physical and biological properties, hydrogels with different functions have been developed and utilized in a broad range of applications, from tissue engineering to soft robotics. However, conventional hydrogels usually suffer from weak mechanical properties and they are easily deformed or damaged when they are subjected to mechanical forces. The accumulation of the damage may lead to the permanent structural change and the loss of the functional properties of the hydrogels. Therefore, it is important to develop mechanically robust hydrogels with autonomous self-healing property in order to extend their lifespan for various applications. In this mini review, we focus on the discussion about the appropriate molecular design of the hydrogel network for achieving self-healing and excellent mechanical properties, respectively as well as the corresponding self-healing and toughening mechanisms. We conclude with perspectives on the remaining challenges in the field as well as the recommendations for future development.

## Introduction

Hydrogels are three-dimensional networks consisting of cross-linked polymer chains, which form matrices with high water content (Annabi et al., 2014). Over the past few years, significant progress has been achieved in the development of functional hydrogels with tunable chemical, physical and biological properties for various applications, including tissue engineering (Eke et al., 2017; Wang J. et al., 2017), controlled drug delivery systems (Zhang et al., 2011; Chen et al., 2018), sensors and actuators (Lei et al., 2017; Yuk et al., 2017), soft electronics (Lin et al., 2016; Cai et al., 2017), etc. However, conventional hydrogels usually suffer from poor mechanical properties and are easily deformed or damaged when they are subjected to mechanical forces. The propagation and accumulation of the damage would affect the integrity of the hydrogels and cause the loss of the functionalities, which may limit the lifespan of the hydrogels. Therefore, it is desirable to develop mechanically robust hydrogels possessing the self-healing capability in order to not only prolong the life-time of the materials but also increase the durability and the reliability of the hydrogels in certain applications by avoiding accumulation of cracks or damages (Graphical Abstract).

**Graphical Abstract:**
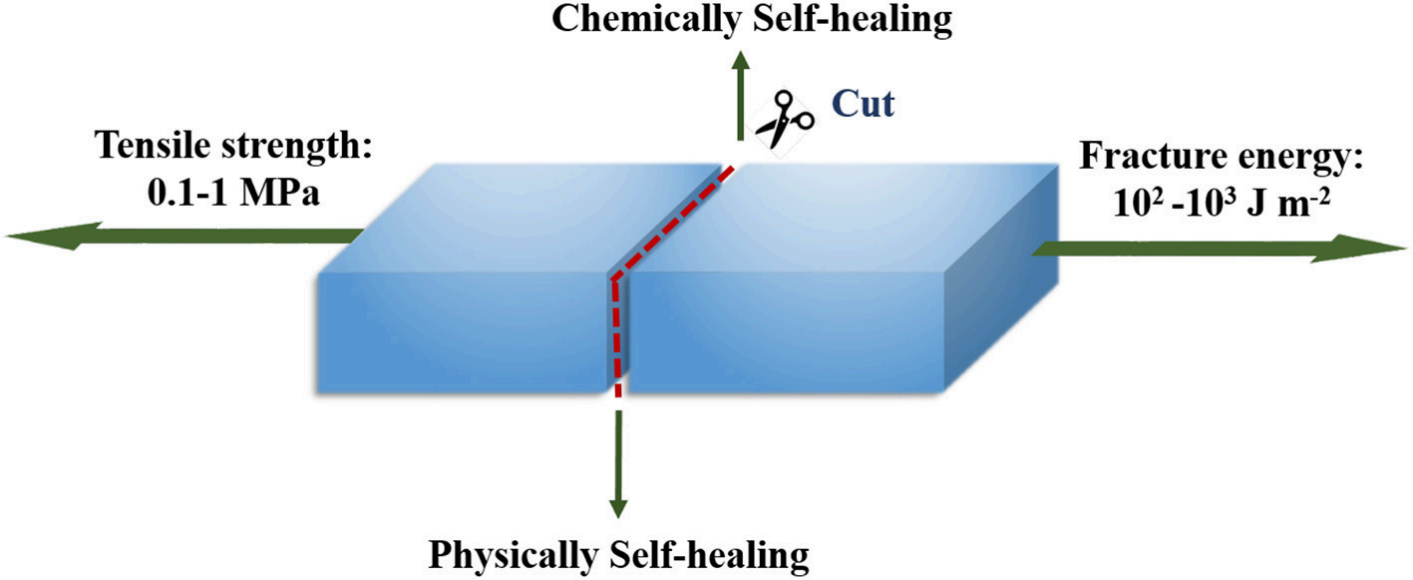
Design of self-healing tough hydrogel.

Self-healing hydrogels are the hydrogels which can automatically heal damages and restore themselves to normality without the intervention of an external stimuli (Taylor and In Het Panhuis, 2016). Based on different self-healing mechanisms, the self-healing hydrogels can be divided into two categories, chemically and physically self-healing hydrogels. Different design strategies can be employed for fabricating hydrogels with self-healing ability. Chemically self-healing hydrogels reform the network through dynamic covalent bonds or reversible chemical reactions, commonly including phenylboronic ester bonds (Wang et al., 2016; Guo et al., 2017), dynamic imine bonds (dynamic Schiff base) (Zhao et al., 2017; Liu et al., 2018), disulfide bonds (Yang X. et al., 2017; Yu et al., 2017), reversible radical reaction (Ida et al., 2016), Diels-Alder reaction (Shao et al., 2017), etc. Physically self-healing hydrogels re-establish the structure through dynamic non-covalent interactions such as hydrogen bonding (Ren et al., 2017), hydrophobic interaction (Xiong et al., 2017), host-guest interaction (Zhang M. et al., 2012), metal-coordination (Andersen et al., 2018), or a combination of multiple intermolecular interactions (Liao et al., 2017; Shao et al., 2018). For both types of self-healing hydrogels, special functional groups are required to be incorporated into the polymer chains for desirable chemical reactions or physical interactions, which mediate the self-healing process, to occur in the damaged region.

Conventional hydrogels are considered to be mechanically weak (toughness <10 J m^−2^), which limits certain applications such as tissue engineering (cartilages or tendon), since these tissues are natural hydrogels possessing high toughness (1,000 J m^−2^) and high tensile strength (30 MPa) (Taylor and In Het Panhuis, 2016). The brittleness (low toughness) of the conventional hydrogels comes from two reasons. Conventionally, hydrogels are synthesized by free radical polymerization of water-soluble monomers and cross-linkers. Due to the difference in reactivity between the reagents, highly random (heterogeneous) cross-linked network is typically produced. The heterogeneity will lead to uneven stress distribution and stress localization when the hydrogel experiences force loading, which accounts for the brittleness (Naficy et al., 2011). Besides, the conventional hydrogels lack of mechanisms which allow the mechanical energy to be dissipated through the hydrogel upon force loading, which is another reason for the brittleness (Chung et al., 2017). It is generally agreed that the hydrogels with tensile strength of 0.1–1 MPa and fracture energy of 10^2^-10^3^ J m^−2^ can be considered as tough hydrogels (Chen et al., 2016). To improve the mechanical properties of the conventional hydrogels, one can either increase the homogeneity of the cross-linked network [tetra-PEG hydrogels (Ishii et al., 2017; Yang N. et al., 2017), radiation cross-lined hydrogels (Xu et al., 2018)] or design the hydrogels with special structure (double network (DN) hydrogels (Li et al., 2016; Liu and Li, 2016), nanocomposite hydrogels (Jiang et al., 2017; Qin et al., 2017)etc., which allows mechanical energy to be dissipated through the hydrogel.

In this mini-review, we briefly discuss different design strategies for developing self-healing and tough hydrogels, respectively. Examples from recent literature will be illustrated accordingly. Finally, we conclude with a perspective on remaining challenges in the field and the recommendations for future development.

## Design strategies for self-healing hydrogels

### Chemically self-healing hydrogels

**Figure 1:**
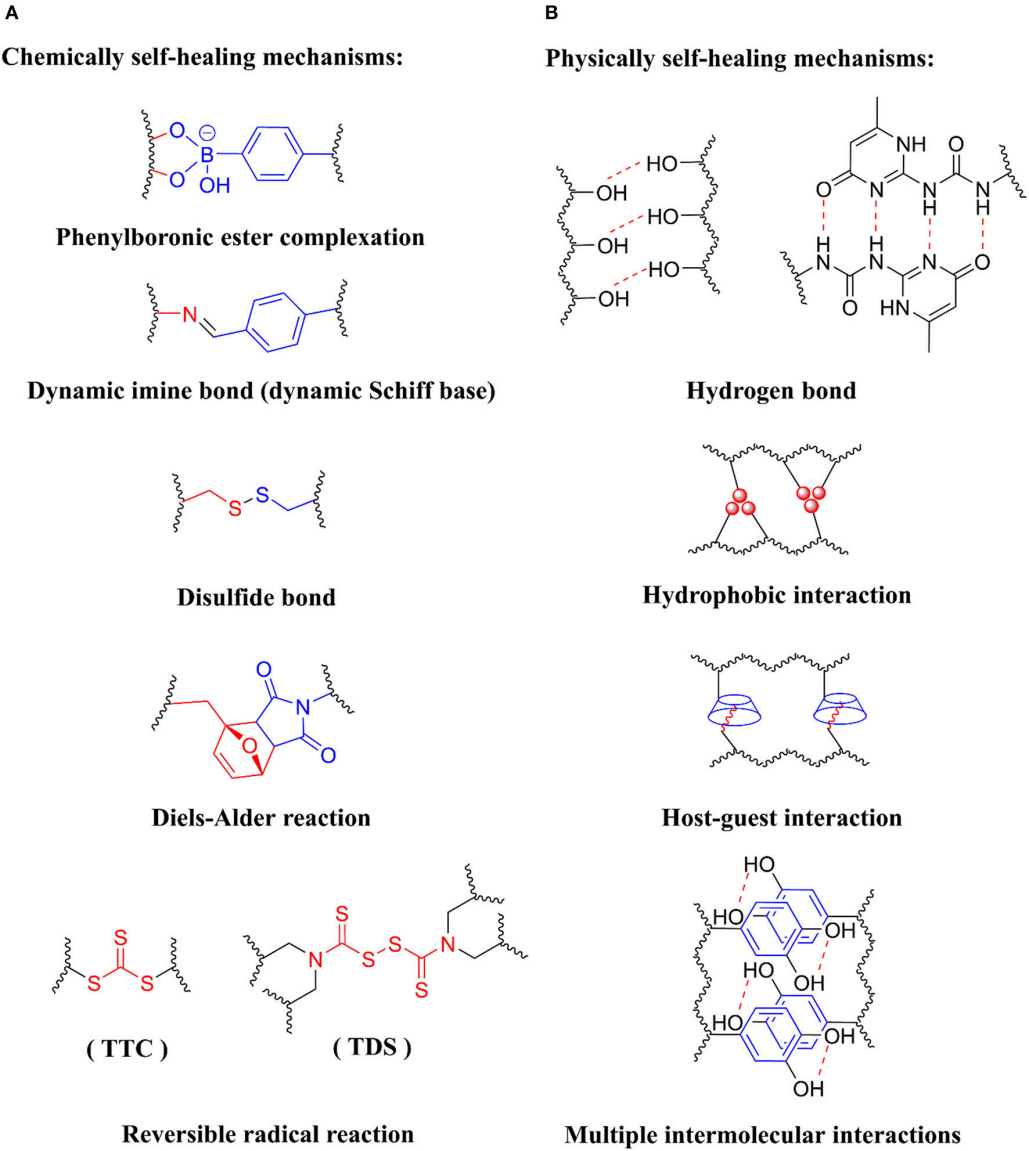
Different strategies for designing self-healing hydrogels. **(A)** Chemically self-healing mechanisms, including phenylboronic ester complexation, dynamic imine bond (dynamic Schiff base), disulfide bond, Diels-Alder reaction and reversible radical reaction, **(B)** Physically self-healing mechanisms, including hydrogen bond, hydrophobic interaction, host-guest interaction and multiple intermolecular interactions.

In conventional hydrogels, the polymer networks are usually cross-linked by covalent bonds which are irreversible and too stable for dynamic chemistry to occur for self-healing (Wei et al., 2014). The chemically self-healing hydrogels reform the network through dynamic covalent bonds or reversible chemical reactions, typically including phenylboronic ester bonds, dynamic imine bonds (dynamic Schiff base), disulfide bonds, reversible radical reaction, Diels-Alder reaction, etc. (Figure 1A).

#### Phenylboronic ester complexation

Phenylboronic ester is the dynamic complex formed based on boronic acid-diol interaction. The dynamic nature (self-healing ability) of the complexation strongly depends on the pK_a_value of various phenylboronic acids. Langer et al. reported an injectable self-healing glucose-responsive hydrogel which was constructed by phenylboronic acid-terminated four-armed poly(ethylene glycol) (PEG) and diol-terminated four-armed PEG (Yesilyurt et al., 2016). It was found that the self-healing behavior and the mechanical property of the hydrogels are closely associated with the pK_a_value of the terminal phenylboronic acid group. When the hydrogel was formed at pH greater than the pK_a_value of the terminal phenylboronic acid group, the gel was rigid and brittle and the self-healing ability was found to decrease with increasing the pH. However, when the hydrogel was formed around the pK_a_ value of the terminal phenylboronic acid group, the hydrogels showed excellent self-healing ability. When the pH is below the pK_a_ value, there is no gel formation observed. The stability (self-healing ability) of phenylboronic ester complex also depends on the conformation of the diol groups in the polymer chains. Schiller et al. determined binding constants between phenylboronic acid and different polysaccharides with different diol conformations (Axthelm et al., 2017). It was found that phenylboronic acid has a higher affinity for the *cis*-diol groups than that for the *trans*-diol groups. Therefore, the self-healing ability of the hydrogel is expected to be enhanced when *cis*-diol is incorporated into the polymers used to construct hydrogel network since the boron-*cis*-diol complexation is more thermodynamically stable and more likely to be reformed after being destructed. The self-healing hydrogels which based on phenylboronic ester complexation have been applied to a wide range of biomedical applications. Narain et al. developed a series of self-healing hydrogels for applications such as glucose sensing (Kotsuchibashi et al., 2013), drug delivery (Chen et al., 2018) and 3D cell cultivation (Wang et al., 2016).

#### Dynamic imine bond (schiff base)

Dynamic imine bonds (dynamic Schiff base) are formed between the aldehyde groups and the primary amine groups. Wei et al. reported a series of self-healing hydrogels based on dynamic Schiff base during the past few years (Zhang et al., 2011; Tseng et al., 2015; Hsieh et al., 2017; Yang X. et al., 2017; Zhang Y. et al., 2017). In most of their work, a benzaldehyde-difunctionalized poly(ethylene glycol) (DF-PEG) was synthesized. Self-healing hydrogels with different properties and applications can be constructed by simply mixing the DF-PEG solution with either natural polymer solutions or synthetic polymer solutions such as glycol chitosan solution (Zhang et al., 2011), amine-modified carboxyethyl cellulose solution (Yang X. et al., 2017), etc. Such self-healing hydrogels can be used in wide biomedical applications such as 3D cell cultivation (Zhang Y. et al., 2017), controlled release of biomolecules (Zhang et al., 2011), blood capillary formation (Hsieh et al., 2017), central nervous system repair (Tseng et al., 2015), etc. Guo et al. also developed self-healing hydrogels which employs dynamic Schiff base for biomedical applications such as cutaneous wound healing (Zhao et al., 2017), cell delivery carrier for cardiac cell therapy (Dong et al., 2016) and drug delivery carrier for hepatocellular carcinoma therapy (Qu et al., 2017). Interestingly, similar to the phenylboronic ester complex, the self-healing ability of the hydrogels based on dynamic Schiff base is also pH dependent. Therefore, appropriate pH condition needs to be met when constructing the hydrogel with desirable self-healing property.

#### Disulfide bond

Disulfide bond is another dynamic covalent bond which is often adopted to make the hydrogel self-healable. The dynamic feature of the disulfide bond is based on the thiol/disulfide exchange reactions which are sensitive to heat, photo-irradiation and mechanical stress. Waymouth et al. developed a self-healing hydrogel which was constructed by an ABA triblock copolymer (Zhang and Waymouth, 2017). The A-block of the polymer contains pendent dithiolane groups which can be cross-linked through reversible ring opening polymerization induced by disulfide exchange reactions between the 1,2-dithiolanes and dithiols. The as-prepared hydrogel underwent reversible sol-gel transition in the presence of external stimuli such as temperature and pH. The hydrogel was able to restore itself after rheological deformation up to a strain value of 800%. Such hydrogel is a promising candidate for stimuli-responsive drug delivery. Wang et al. developed a self-healing hydrogel coating which based on disulfide bond with antibacterial and antifouling properties (Yang et al., 2015). The as-prepared hydrogel coating has great potential as an effective and durable coating in long-term applications for biomaterials.

#### Other dynamic chemical bonds and reactions

Diels-Alder reaction is one of the “click chemistry” which occurs between a diene and a dienophile (Gregoritza et al., 2017; Guaresti et al., 2018). Yang et al. reported a self-healable cellulose nanocrystal-poly(ethylene glycol) (CNC-PEG) nanocomposite hydrogel via Diels-Alder reaction. The hydrogel possessed both excellent self-healing property and mechanical properties, which can be potentially used for tissue engineering (Shao et al., 2017). Chen et al. reported that the self-healing hydrogel based on Diels-Alder reaction was completely self-healed after 7 h (Wei et al., 2013). For the self-healing hydrogels based on the reversible radical reaction, functional groups such as trithiocarbonate (TTC) units (Amamoto et al., 2011), thiuramdisulfide (TDS) units (Amamoto et al., 2012), etc., need to be integrated into the polymer network. The reversible radical reaction can be triggered by external stimuli such as photo irradiation for the hydrogels to be self-healed (Wei et al., 2014).

### Physically self-healing hydrogels

This section focuses on designing physically self-healing hydrogels based on dynamic non-covalent interactions, including hydrogen bonds, hydrophobic interaction, host-guest interaction and multiple intermolecular interactions (Figure 1B).

#### Hydrogen bond

Hydrogen bonding is one of the most commonly used strategies to construct physically self-healing hydrogels. Zhao et al. prepared a poly(vinyl alcohol) (PVA) hydrogel through facile freezing/thawing method (Zhang H. et al., 2012). It was found that the hydrogel showed excellent self-healing behavior only when the polymer concentration is >35 wt %, which indicates that sufficient number of hydroxyl groups are required to promote the regeneration of enough hydrogen bonds across the interface for the hydrogel to be self-healed. The conformation of hydrogen bond donors and acceptors is another factor which may affect the formation of hydrogen bonds and influence the self-healing performance. Wang et al. designed a self-healing hydrogel which contains ureido pyrimidinone (UPy) moieties (Zhang G. et al., 2017). The UPy moieties can be dimerized through quadruple hydrogen bonds. The hydrogel showed excellent self-healing performance due to the multiple hydrogen bonds formed in the network. Gu et al. prepared a self-healing hydrogel which was formed through hydrogen bonding between the cytosine (C) and guanosine (G) modified hyaluronic acid (HA) (Ye et al., 2017). The as-prepared hydrogel could be applied for short-term injectable drug delivery, tissue engineering and regenerative medicine.

#### Hydrophobic interaction

Hydrophobic interaction serves as another strategy to design physically self-healing hydrogels. In these hydrogels, hydrophobic monomers are incorporated into the hydrogel network together with the hydrophilic monomers. Hydrophobic associations or hydrophobic domains will be formed within the hydrogel through self-assemble. By carefully controlling the hydrophobe content in the hydrogel, the resulting hydrogel can be self-healed through strong hydrophobic interactions. Okay et al. reported several self-healing hydrogels which were based on hydrophobic interactions and the resulting hydrogels showed excellent self-healing properties (Tuncaboylu et al., 2011; Mihajlovic et al., 2017). Owusu-Nkwantabisah et al. developed a thermoresponsive self-healing hydrogel which incorporated poly(N-isopropylacrylamide) (PNIPAM) nanogels (Owusu-Nkwantabisah et al., 2017). The as-prepared hydrogel showed rapid self-healing behavior through hydrophobic interaction upon raising the temperature. Such hydrogel can be potentially applied for temperature sensors, thermo-switchable windows in biomedical applications.

#### Host-guest interaction

Host-guest interaction is one of the supramolecular interactions which is also widely employed to construct physically self-healing hydrogels. The interaction usually involves two or more molecules forming complex through various dynamic non-covalent interactions such as hydrogen bonds, electrostatic interactions, van der Waals forces, etc., (Wei et al., 2014). Typical examples of the host molecules including, cyclodextrin (CD) (Loebel et al., 2017), cucurbituril (CB) (Xu et al., 2017), crown ether (CE) (Zhang M. et al., 2012), etc., which have been used to make self-healing hydrogels for different applications. The internal cavity of these molecules allows them to accommodate various guest molecules with different binding affinity (Nakahata et al., 2011). Therefore, one should carefully choose the host-guest pair since the self-healing ability strongly depends on the binding affinity between the host and guest molecules. Guo et al. developed several conductive self-healing hydrogels which are based on host-guest interaction. Such hydrogels can be potentially used for a wide range of biomedical applications where electroactivity is in need such as bio sensors (Deng et al., 2018) and carriers for therapeutic agents (Wu et al., 2014).

#### Multiple intermolecular interactions

In order to achieve shorter self-healing time and higher self-healing efficiency, multiple intermolecular interactions are employed to endow the hydrogel with self-healing property. Mussel-inspired self-healing hydrogels are typical examples which contain polydopamine (PDA) within their structure. PDA based materials are well known for their remarkable adhesive property through synergestic effect of various interactions such as hydrogen bonds, metal-catechol coordination, cation–π interactions, π-π interactions, etc., (Li et al., 2015b). Zeng et al. reported several mussel-inspired self-healing hydrogels with antifouling and antimicrobial properties (Li et al., 2015c, 2017). The hydrogels showed excellent self-healing performance and such hydrogels have great potential in wound healing application.

## Design strategies for tough hydrogels

### Homogeneous tough hydrogels

**Figure 2:**
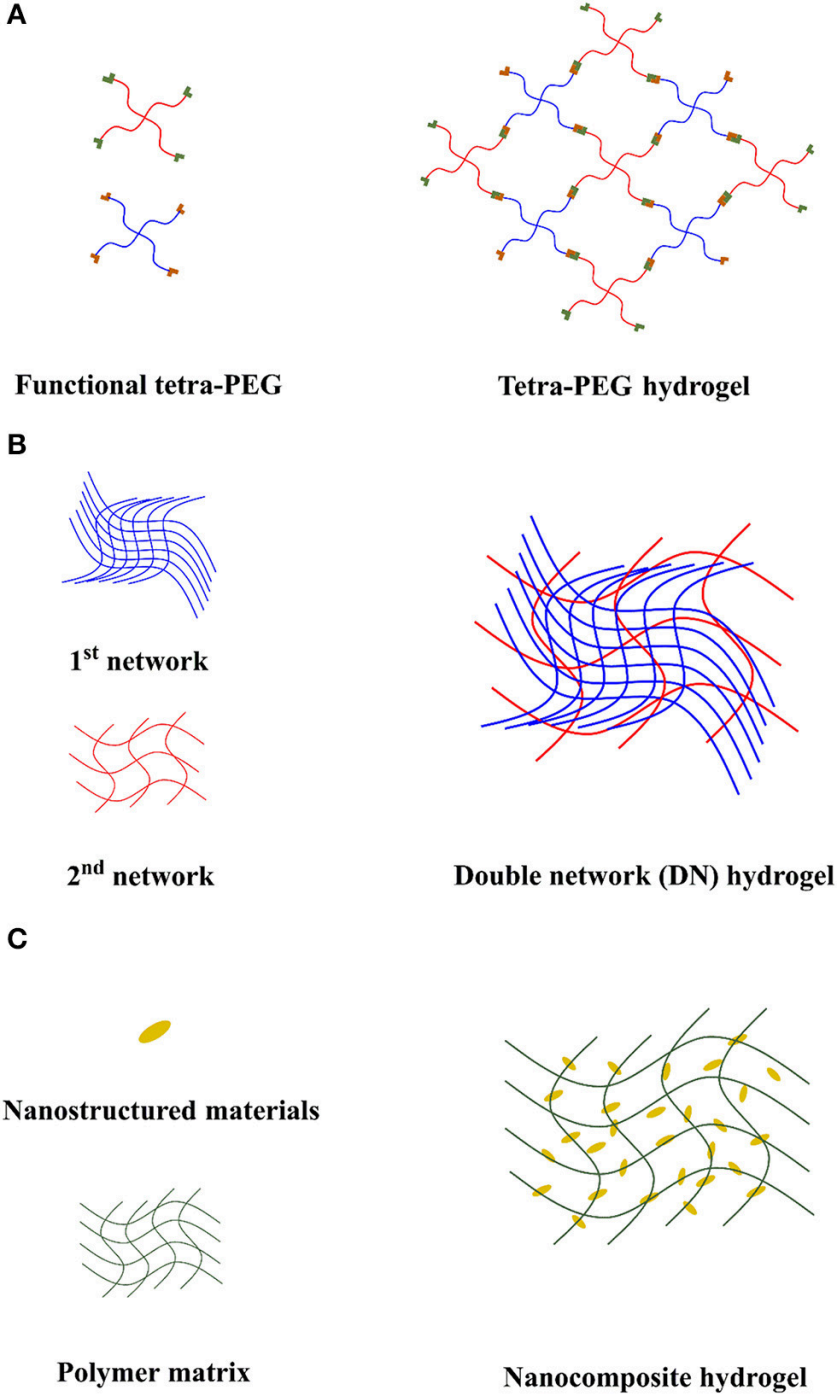
Typical strategies for designing tough hydrogels. **(A)** Tetra-PEG hydrogel, **(B)** double network hydrogel (DN hydrogel), **(C)** nanocomposite hydrogel (NC hydrogel).

Tetra-PEG hydrogel is a typical example of homogeneous hydrogels (Figure 2A). Sakai et al. did pioneering work on developing tetra-PEG tough hydrogel (Sakai et al., 2008). In 2008, the first tetra-PEG hydrogel was developed. Two tetra-PEG macro-monomers with the same arm length and different end group were synthesized. The hydrogel was fabricated by simply mixing two tetra-PEG solutions to allow crosslinking reactions between the end groups. The mechanical property of the developed hydrogel was compared with that of agarose gel and acrylamide gel by compression test. It was found that tetra-PEG hydrogel prepared at 1:1 mole ratio of two macro-monomers experienced the highest compression strength of ~2.5 MPa. The enhanced toughness of the hydrogel results from the homogenously cross-linked network, which can not only avoid stress concentration in certain regions but also allows the stress to be distributed evenly (Naficy et al., 2011).

Homogeneously cross-linked hydrogels can also be fabricated through radiation cross-linking method instead of chemical cross-linking method. Wang et al. synthesized a polyacrylic acid (PAA) and polyacrylamide (PAAm) hydrogel throughγ-radiation-induced polymerization (Wang et al., 2011). It was found that the mechanical property of the PAA-PAAm hydrogel was superior to those produced by the classical chemical polymerization using a crosslinking agent. This may be due to the non-selective initiation effect of the monomers when a high-energy irradiation source is in presence, which could lead to a more homogeneous hydrogel network and improve the mechanical properties of the hydrogel.

### Double-network tough hydrogels (DN hydrogels)

Double-network hydrogels (DN hydrogels) are considered as one class of tough hydrogels which employ mechanical energy dissipation mechanism (Figure 2B). The high strength and toughness of the DN hydrogels are resulted from their unique contrasting network structure and strong network entanglement (Chen et al., 2016). The first network is often designed to be tightly cross-linked and serves as the minor component of the DN hydrogel. The second network is designed to be loosely cross-linked and it is the major component of the DN hydrogel (Gong, 2010). Gong et al. reported the first DN hydrogel in 2003 (Gong et al., 2003), in which the poly(2-acrylamido-2-methylpropanesulfonic acid)/poly(acrylamide) (PAMPS/PAM) DN hydrogel showed fracture stress of 17.2 MPa which is 20 times higher than that of either PAMPS or PAM single network hydrogel. Brown and Tanaka proposed the toughening mechanism of the DN hydrogel (Brown, 2007; Tanaka, 2007). During the fracture process, the tightly cross-linked PAMPS network is firstly fragmented into micro-clusters and they act as the cross-linking points which are able to hold and stabilize the loosely cross-linked PAM network. The brittle PAMPS network serves as the “sacrificial bonds” which allows energy to be dissipated during the formation of the micro-cracks. However, the largest disadvantage of the DN hydrogel developed by Gong et al. is that the brittle PAMPS network is irreversibly cross-linked and the fracture of the PAMPS network is permanent. Such DN hydrogel will lose its toughness once the first polymer network is ruptured. Later, Suo et al. reported a DN hydrogel which composed of a dynamically cross-linked alginate network based on the ionic interactions between Ca^2+^ and carboxylic groups (COOH^−^) on the alginate as the first “sacrificial network” and a chemically cross-linked PAM network as the second network (Sun et al., 2012). The DN hydrogel not only showed superior mechanical property but also recovered most of its mechanical properties after first force loading. Due to the excellent mechanical properties, DN hydrogels attract great interest in biomedical applications. Guo et al. developed biocompatible functional hydrogels with excellent mechanical properties for potential applications of tissue implantation (Li et al., 2015a) and drug delivery (Zhao et al., 2014).

### Nanocomposite hydrogels (NC hydrogels)

Nanocomposite (NC hydrogels) are the hydrogels formed between nano-scaled materials and polymer chains (Figure 2C). The concept of NC hydrogel was established by Haraguchi and Takehisa (2002). In this work, they developed a novel NC hydrogel which contains clay and poly(N-isopropyl acrylamide) (PNIPAM) chains. The hydrogels was formed by *in-situ* free radical polymerization of the NIPAM monomers in well-dispersed clay aqueous solution without using chemical cross-linkers. A mechanism was proposed for the hydrogel formation: First, the initiator could bind to the surface of clay through electrostatic interactions which allows polymer chains to be grafted from the clay nanosheets. In addition, the nucleophilic N(H)CO in PNIPAm chains could coordinate with the Si on the clay. Therefore, the clay nanosheets can act as multifunctional cross-linkers which allow multiple polymer chains to be attached through various interactions. The clay/PNIPAM NC hydrogels possessed excellent mechanical properties with tensile stress of 0.109 MPa and elongation ratio of 1,424%. The outstanding mechanical properties of the NC hydrogel is due to the synergistic effect of the multiple reversible intermolecular interactions between nanomaterials and polymer chains for mechanical energy dissipation (Haraguchi, 2007) and the homogeneity of the well-dispersed nanomaterials within the hydrogel network for even distribution of stress (Chung et al., 2017).

It is worth mentioning that through special design of NC hydrogels, one can fabricate hydrogels with both outstanding mechanical properties and excellent self-healing properties. Lu et al. adopted mussel-inspired surface chemistry to modify the surfaces of the nanomaterials with polydopamine (PDA) and developed a series of tough self-healing NC hydrogels for various applications such as reduced graphene oxide (rGO)/polyacrylamide (PAM) NC hydrogels for implantable bioelectronics (Han et al., 2017b), nanoclay/PAM NC hydrogels for wound healing (Han et al., 2017a), carbon black nanoparticle (CBNP)/PAM NC hydrogels for electricity conduction (Han et al., 2017d), etc. With the modification of PDA, multiple reversible intermolecular interactions from PDA are introduced into the hydrogel system, which not only improve the mechanical properties but also endow the hydrogel with self-healing capability. Similarly, Shao et al. developed a tough self-healing NC hydrogel which based on tannic acid-coated cellulose nanocrystals (TA@CNCs) for wearable strain sensor (Shao et al., 2018). The TA@CNCs act as dynamic bridges in the hydrogel network, which lead to rapid self-healing performance as well as reliable mechanical properties of the as-prepared hydrogel.

## Concluding remarks

Mechanically robust hydrogels with self-healing properties present many advantages over conventional hydrogels as they can be utilized in a broad range of areas such as stretchable electronics (Pu et al., 2017; Jing et al., 2018; Hong et al., 2019), sensors and actuators (Francis et al., 2017; Tudor et al., 2017), cartilage repair (Han et al., 2017c; Lolli et al., 2018), with extended life-time and enhanced durability and reliability. Although the field of study shows great promise, there are still challenges to be tackled.

Generally, the self-healing ability of the hydrogels weakens and the time required for the healing process increases with the improvement of the mechanical properties of the hydrogels as summarized by Panhui et al. (Taylor and In Het Panhuis, 2016). Therefore, the development of mechanically robust hydrogels which can be self-healed on the time scale of seconds or minutes should be considered. One possible approach is to introduce multiple chemically or physically self-healing mechanisms into the NC hydrogels/DN hydrogels in order to enhance the self-healing efficiency without sacrificing the mechanical properties.

The fundamental understanding of different self-healing and toughening mechanisms is crucial for designing and optimizing the self-healing tough hydrogels. Both theoretical and experimental studies are needed to understand the self-healing and toughening mechanisms at the atomic-scale or molecular-scale. For instance, Zeng et al. used a surface forces apparatus (SFA) to quantitatively study the polymer interactions which account for the self-healing behavior of the hydrogel (Yan et al., 2017). Such non-destructive *in situ* testing method is highly needed for elucidating the molecular interaction mechanisms of various hydrogel materials.

It is also necessary to develop self-healing tough hydrogels into multifunctional “smart” materials, which should integrate other properties such as electrical conductivity (Wu et al., 2017), temperature responsivity (Zubik et al., 2017), photo responsivity (Wang R. et al., 2017), etc., into a single hydrogel system. Such hydrogels can be promisingly applied in different applications such as sensors and actuators. When the hydrogels are endowed with the stimuli responsive properties, one should ensure the chemical and mechanical stability of the hydrogels as the change of environmental conditions may affect the intrinsic properties of the hydrogels. The intermolecular and surface interactions of various components in the hydrogel systems essentially determine the properties and performances of the hydrogel systems, which should be well taken in the rational design of the self-healing tough hydrogels.

## Author contributions

WW wrote the manuscript. RN and HZ helped to revise the manuscript.

### Conflict of interest statement

The authors declare that the research was conducted in the absence of any commercial or financial relationships that could be construed as a potential conflict of interest.
